# A ‘game of dressings’: Strategies for wound management in primary health care

**DOI:** 10.4102/safp.v64i1.5462

**Published:** 2022-02-28

**Authors:** Maria G.C. Giaquinto-Cilliers, Arun Nair, Klaus B. von Pressentin, Francois Coetzee, Hamid Saeed

**Affiliations:** 1 Department of Plastic and Reconstructive Surgery, Faculty of Medicine, University of the Free State, Bloemfontein, South Africa; 2 Department of Plastic and Reconstructive Surgery, Robert Mangaliso Sobukwe Hospital, Kimberley, South Africa; 3 Department of Family Medicine, Faculty of Medicine, University of the Free State, Bloemfontein, South Africa; 4 Department of Family Medicine, Robert Mangaliso Sobukwe Hospital, Kimberley, South Africa; 5 Division of Family Medicine, School of Public Health and Family Medicine, Faculty of Health Sciences, University of Cape Town, Cape Town, South Africa; 6 Department of Global Health, Ukwanda Centre for Rural Health, Faculty of Medicine and Health Sciences, Stellenbosch University, Bellville, South Africa; 7 Department of Family Medicine, Faculty of Family Medicine, University of the Free State, Bloemfontein, South Africa

**Keywords:** strategies, wound care, wound management, wound-bed-preparation, dressings, framework

## Abstract

Wound care management and the dressing of wounds represent some of the most basic services offered in primary health care facilities across Southern Africa. Clinicians should have a basic understanding of the processes of wound healing and wound bed preparation to match the ideal cost-effective dressing to the particular type of wound to be managed. In the ‘kingdom of wounds’, the authors use a popular analogy and propose the best five strategies for the clinician in choosing the right management option in the ‘game of dressings’.

## Introduction

The broad definition of a wound is that it is a breach in the integrity of skin, derived from different aetiology and extent, resulting in the breakdown of its protective functions.^1^ Wounds may be classified as acute or chronic based on the source or causal agent, including surgical wounds, burns, traumatic wounds, pressure injuries, venous stasis ulcers, arterial ulcers, diabetic foot ulcers and other atypical chronic wounds. Chronic wounds affect around 8.2 million people in the United States with an estimated cost of between 28 billion dollars and 31 billion dollars.^2^ There is no current estimate in literature for the number of people affected in South Africa or the associated costs, with very few international studies roughly estimating it.^3^

The market of wound dressings in South Africa is moderately competitive with some major players and smaller companies launching new branded products every year. The global projected growth of the advanced dressings market is 6.4% over six years from 2021.^4^ Several factors are driving the increased need for efficient wound care, amongst them a demand for faster recovery of patients with wounds; the need for reduction of hospital stay; the rising incidence of chronic diseases that can result in wounds (diabetes, cancer, autoimmune diseases) and the expected increase in the number of surgical procedures.^4^ Efficient wound care significantly reduces the cost of care; the burden of disease on healthcare facilities and improves patient outcomes.^5^ The active ingredients used by several products may be similar when comparing products from different companies, but the price of those products may vary with higher prices usually offered by the major player companies that have more marketing power. High-cost products have no clinical benefit compared with low-cost products with similar active ingredients.^6^ This diversity of products may be daunting for clinicians and healthcare workers, which can lead to the prescription of dressings not indicated for a particular wound resulting in a lack of progress towards the healing of stalled wounds and unnecessary costs for the patients and or health facilities.

Wound care management and dressing of wounds represent some of the most basic services offered in the primary healthcare clinics across South Africa yet in a study carried-out in South Africa in 2010, it was found that undergraduate students who left medical school were not equipped with the necessary knowledge to treat chronic wounds.^7^ Therefore there is a need to educate healthcare practitioners who are providing this basic service and to optimise the indication of dressings for wound care. Treating clinicians should have a basic understanding of the processes of wound healing and wound bed preparation (WBP) to choose the ideal cost-effective dressing to apply to the particular type of wound to be managed. This is applicable until complete healing is achieved either by spontaneous healing, using adjuvant therapy or using additional surgical procedures.

The *Game of Thrones* is a fantasy drama television series that was aired in the United States in April 2011.^8^ The series was a big success around the world, with a legion of fans choosing their ‘houses’ amongst the ‘Seven Kingdoms’ in the fight for the ‘Iron Throne’. In the ‘kingdom of wounds’, particularly chronic wounds, companies that manufacture dressings for wound care are competing in a market that drives the cost of care in the sphere of billions of dollars.^6^ The targeted ‘throne’ of this competition is the prescribing physician and/or the healthcare professional who is taking care of patients with wounds, usually a nurse under the supervision of the physician’s orders.

Five strategies are offered in the following subsections to guide clinicians in their choices for dressings that are applicable in both hospital settings and primary healthcare settings.

### Strategy number 1: Know the type of wound to be treated

Wounds may occur during many diseases and traumatic injuries, with types varying according to the source of the causal agent. Wound healing is a complex series of interlinked and co-dependent events that consist of haemostasis, coagulation, migration of cells, inflammation, cell proliferation and differentiation, matrix repair, epithelialisation and remodelling of the scar tissue.^9,10^ This process involves a balance between growth factors, pro-inflammatory cytokines and their inhibitors; matrix metalloproteinases (MMPs) and their inhibitors.^10^ All these elements are responsible to initiate the inflammatory phase that lasts more or less than for4 days (with a peak 5–7 days after initial injury). This is followed by the repair phase with proliferation and migration of fibroblasts, production of collagen and neovascularization. There is a migration of epithelial cells from wound edges and cells niches in hair follicles and sweat glands. This is followed by the contraction of the wound because of the differentiation of fibroblasts into myofibroblasts. This initial scar matrix is then remodelled for the next 6–12 months.^10^

In acute wounds (i.e. post-surgery and post-traumatic), the healing process happens in an orderly and timely fashion, while in chronic wounds (i.e. pressure injuries, leg ulcers and diabetic foot ulcers), there is a ‘stop’ in the orderly healing process because of a lack of cell division, increased levels of pro-inflammatory cytokines (resulting in excess inflammation), an imbalance and increase in the levels of MMPs causing degradation of tissue and growth factors and the cells becoming senescent.^10,11^ Consequently, the wound becomes ‘stuck’ in the inflammatory and proliferative stages of healing, taking more than 6 weeks to heal.^12^

If no systemic (e.g. nutritional status, anaemia, chronic diseases) or local factors (e.g. presence of a foreign body, haematoma/seroma, infection, wound dehiscence) cause a delay in the wound healing process, a wound is supposed to heal by epithelial regeneration, primary closure or primary intention. However, in the presence of systemic or local factors that delay wound healing, where possible these factors should be addressed by the appropriate intervention, for example, correcting the nutritional status and anaemia, removing the foreign body or treatment of wound infection. Where local factors cannot be ameliorated such as wound dehiscence, wounds may be left to heal by secondary intention or spontaneous healing, followed by delayed closure or tertiary intention.^13,14^

### Strategy number 2: Know the healability of the wound to be treated

**FIGURE 1:**
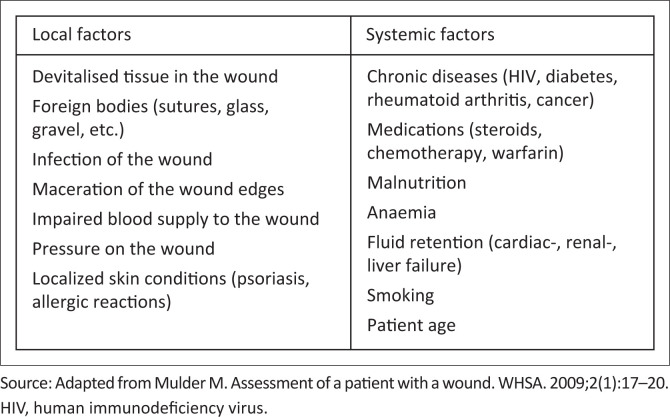
Common local and systemic factors that may delay wound healing.

A wound is considered *healable*:

- If the cause provoking the wound is known and treatable.
- If there is an adequate blood supply to assist in healing.
- If there are no systemic factors that prevent healing: co-existing medical conditions, nutritional status, use of medications that prevent healing or factors impeding the patient to receive adequate management^15^ (see Figure 1^16^).

Some wounds are referred to as *maintenance* wounds when the cause of the wound for some reasons cannot be treated, but the blood supply is adequate. In other words, if other factors can be managed, the wound will probably heal. Maintenance wounds may represent a stage during the treatment, where the wound is stalled in the progression for healing; the physician should then reassess the systemic factors affecting the healing progress. *Non-healable* or *palliative* wounds imply that all three factors above are affected, that is, the cause cannot be treated, blood supply is often inadequate and co-factors may inhibit healing.^15^ Examples of wounds where palliation or maintenance could be the treatment goal include bedbound patients with very poor nutritional status and are unfit for surgery, patients with advanced human immunodeficiency virus (HIV) disease that has a poor prognosis, patients with metastatic cancer receiving palliative care or advanced cancer wounds. It is important to recognise these factors to decide what has to be changed in the treatment plan and initiate the early involvement of a multidisciplinary team to make a proper assessment of correctable factors that stall the progression to healing.^17^ This will prevent unnecessary costs and unnecessary wastage of expensive dressings.

### Strategy number 3: Assess and prepare the wound bed

Wound bed preparation is the ‘management of a wound to accelerate endogenous healing or to facilitate the effectiveness of other therapeutic measures’.^18,19^ Frameworks or enablers have been proposed with structured approaches to wound management to help clinicians optimise conditions at the wound bed aiming to reach wound healing.^12^ The most popular WBP frameworks are the acronyms TIME (tissue, inflammation/infection, moisture, edge) and DIME (debridement, inflammation/infection, moisture, edge).

*TIME* framework (Figure 1) has four components: *T*issue management; control of *I*nfection and *I*nflammation; *M*oisture balance and advancement of the epithelial *E*dge of the wound.^12,15,18,19^ An ‘S’ was added later to represent the **s**urrounding skin, currently considered as the peri-wound tissue.^20^

**Tissue** may be:

- Viable – with healthy granulating tissue, usually associated with a bright *reddish* colour or epithelialising tissue, associated with a more *pinkish* colour
- Non-viable – consisting of necrotic tissue or ‘eschar’, the brownish or *blackish* dry tissue in the wound; ‘slough’ tissue, showing a creamy *yellowish* devitalised tissue or infected tissue that is somehow associated with a *greenish* colour often associated with colonisation or infection by *Pseudomonas* species.

For epithelial cells to migrate across the wound surface, a well-built extracellular matrix is required. Hence the need to identify and address the presence of devitalised tissue usually with some form of debridement. Debridement should be conservative if the blood flow to the wound area is affected.^12^ Although surgical debridement is used commonly, other modalities such as chemical debridement, enzymatic debridement and using a water jet can also be utilised for certain wounds.^21^

Wound *infection* can be characterised as a continuum where the progression of infection in a wound aligns with the phases of infection detected in the wound and with the clinical response of the individual to the wound.^22^ The stages within this continuum process include^22,23^:

- *Contamination*: where microbial species will not multiply, and their presence is not detrimental to the wound.
- *Colonisation*: microbial species grow and divide but do not have sufficient levels or virulence to disturb wound healing or immune response where only vigilance is required and no antimicrobials are indicated. All chronic wounds can be regarded as colonised.
- Local infection with *subtle* signs such as hypergranulation, bleeding friable granulation, epithelial bridging and pocketing in granulation tissue, wound breakdown and enlargement, new or increasing pain and malodour and delayed wound healing. The *Classic* signs of local infection are erythema, local warmth, swelling, purulent discharge, new or increasing pain, increasing malodour and delayed healing.
- *Spreading* infection with extending induration or erythema, lymphangitis, wound breakdown, malaise, loss of appetite.
- *Systemic* infection with severe sepsis, septic shock, organ failure and death.

Topical antimicrobials (such as silver-based topical antimicrobials) are indicated from the stage of local infection, and systemic antimicrobials when spreading infection is diagnosed.^23^ The presence of biofilm, that is, a community of micro-organisms surrounded by an extracellular polymeric matrix, may be detected from the stages of local infection and be associated with an increase in the number and virulence of microbial species. Thus, biofilms present in chronic wounds should be treated with appropriate cleaning agents (see dressings classification further below).^10,22^

The formation of an exudate is part of normal wound healing, providing a *moist* wound environment for the proliferation of cells and assisting with the migration and support for the removal of devitalised tissue (autolysis).^12^ When this occurs in excess, it increases the risk for infection, friction damage, maceration of surrounding tissue and an increase in wound size with delayed healing. It is therefore important to note the changes in the exudate consistency (serous, fibrinous, purulent, haemopurulent and haemorrhagic).^12^

For the *edges* to advance (migration of the epidermal cells from the margins across the wound bed), all other factors from the TIME enabler should be optimised. A non-advancing edge may reflect local infection as with undermining, rolling of the edges and pockets in the wound bed.^12^

The *peri-wound* tissue is also assessed regarding maceration (reflecting the excess of exudate), skin integrity (erosions, skin-stripping), induration, oedema, erythema/cellulitis/folliculitis, callous, xerosis, atopic eczema and hyperkeratosis and should be addressed in the treatment plan.^12,20^

**FIGURE 2:**
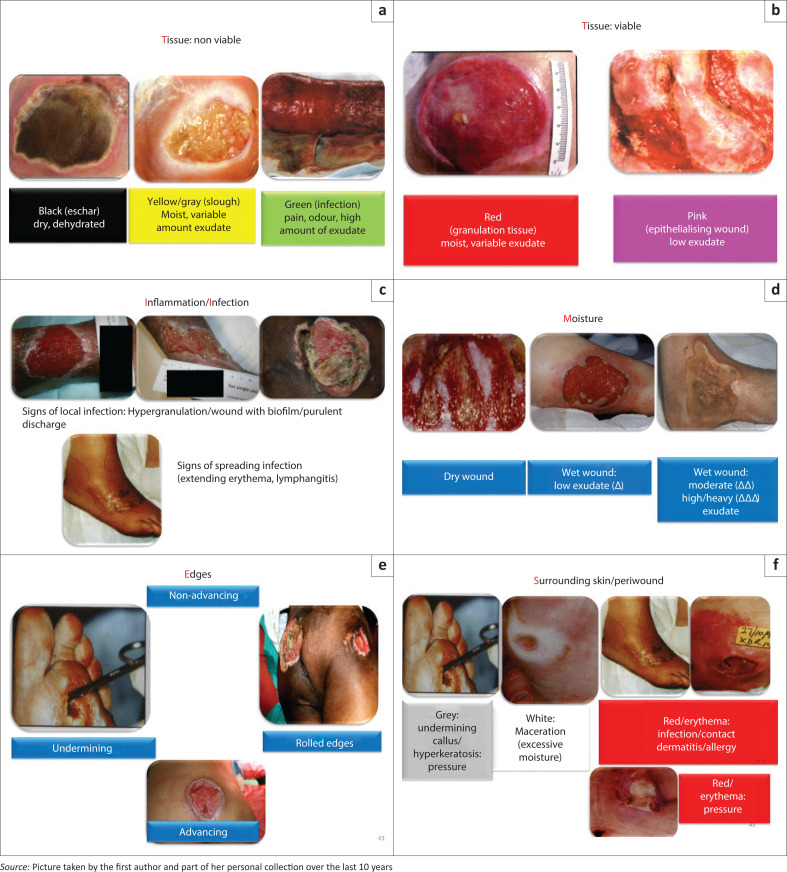
Pictures of examples of tissue (a, b, c), moisture (d), edges (e) and surrounding skin (peri-wound) (f) found in the TIME framework.

The *DIME* framework (Figure 2^15,24^) incorporates the same principles of TIME, adding concepts regarding healability of wounds and the need to address the cause and other factors related to wound management into the enabler; *D* stands for *d*ebridement (surgical, sharp, autolytic, enzymatic, larval or biological and mechanical), **I** for *i*nflammation/*i*nfection, *M* for *m*oisture and *E* for *e*dge.^15,24^

### Strategy number 4: Provide the treatment plan

The frameworks for WBP are tools to be used within the context of the holistic assessment of the patient. Patients need to understand the treatment plan and be involved with the treatment of the underlying cause of the wound for the wound management to be successful.^12^ A holistic approach should include the assessment of the patient’s general health condition, nutritional status, lifestyle habits, current medications as well as the social, economic and psychological factors that may influence wound healing.^11,16^

Wounds must be assessed and documented in terms of the type of wound, location in the body, size and shape with measurements of length, width and depth, amount of exudate, presence of odour/devitalised tissue/signs of inflammation or infection, level of pain, duration and any previous treatment done on the wound. Hence the use of measurement tools such as tape measurements, tracing paper or photography is important to document the progression of treatment.^16,25^

**FIGURE 3:**
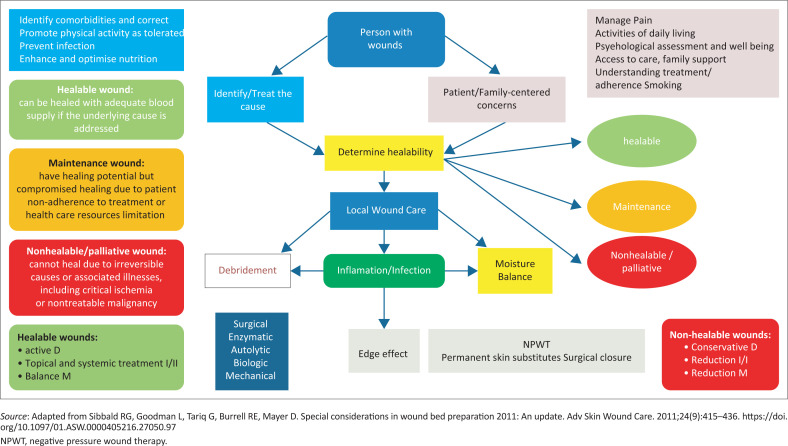
Adaptation of the DIME wound bed preparation framework incorporating the concepts of healability and management of wounds accordingly.

Once the wound bed is assessed, the choice for the dressing will become an easier task (Figure 3^13^).

The treatment plan can be determined as per the WBP framework and should be reassessed at regular intervals. If after 2 weeks of treatment, there are minimal changes in the measurements of the wound in comparison with the initial measurements (edge effect), or if the wound bed shows changes in the type of tissue, levels of exudate/moisture, subtle or classic signs of local infection, non-advancing edges or new wound breakdown, then one can modify or change the treatment plan accordingly.^12,16,17,25^

### Strategy number 5: Choosing the dressing

At any particular time in the assessment of any wound, clinicians should keep in mind that the wound status is an evolving process and adapt their choice of dressings accordingly. There is no single perfect dressing in the market that can be used for all stages of the wound healing process until the complete closure of the wound.

An ideal dressing should have the following characteristics: be easy to apply, safe to be removed without causing skin damage or pain; be cost-effective, stay intact for longer periods, avoid the need for frequent changes; conform to the wound bed/anatomical location, prevent leakage and suit the level of exudate; absorb odour when indicated, control bacterial proliferation and not cause allergic reactions.^20,25,26^

Certain dressings are classified in South Africa as non-invasive medical devices and not medication and some of the advanced dressings need to be prescribed by a physician. The South African Health Products Regulatory Authority (SAHPRA) published in 2014 (updated in 2019), the guidelines for the licensing of manufacturers, distributors and wholesalers, with the registration of medical devices both non-invasive and invasive.^27^ Dressings are classified according to rules and classes depending on what they contain:

- Rule 13, class D: antimicrobials agents.
- Rule 14, class D: viable or non-viable animal tissues or derivatives such as collagen dressings, porcine xenograft dressings
- Dressings that are classified, depending on the intended purpose:
- Rule 1 classes B and C.
- Rule 4 class A.

The guidelines aim to maintain the safety, quality and performance of those classified as medical devices; the classification may differ from other countries.^27^

**FIGURE 4:**
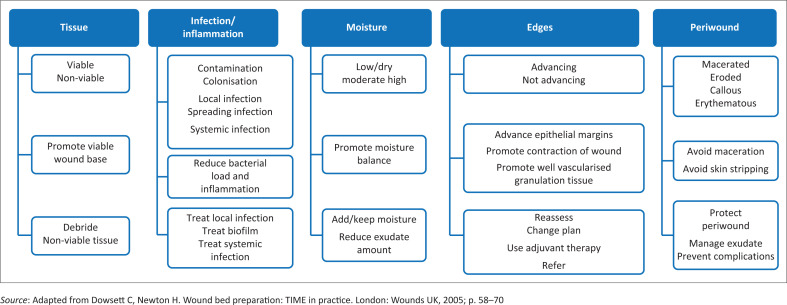
Wound bed preparation and treatment plan: Assessment, objectives and action.

The Wound Healing Association of Southern Africa (WHASA) has published and updated its classification of wound dressings regularly since its conception, because of the addition of new dressings in the South African market.^16,28^ Choosing a particular dressing is a daunting task for the clinician who is not trained in wound management, as some products are listed in more than one category within the classification and the list of dressings available is continually expanding. The latest versions of the classification follow the structure of the WBP framework to facilitate the clinician when faced with new products launched in the market (Figure 4^12,24^).

**FIGURE 5:**
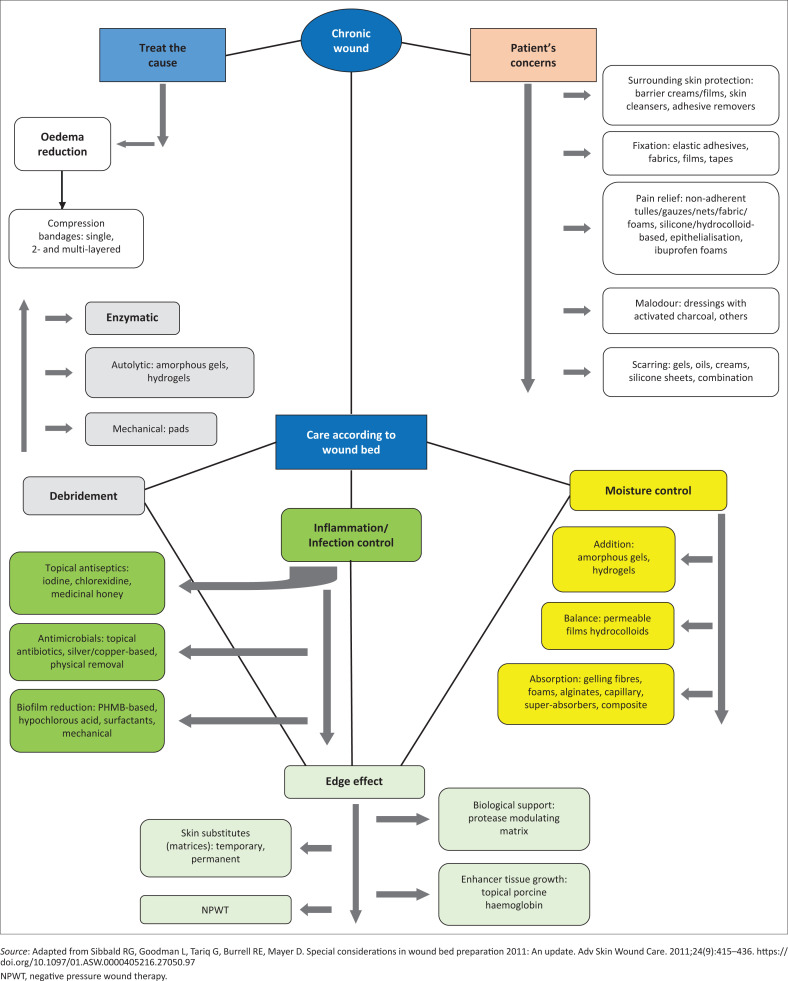
Classification of dressings adapted to the wound bed preparation framework.

When an adequate treatment plan is in place, a healable wound should be 30% (20% – 40%) smaller by week four, to heal by week six.^15,24,26^ Some wounds may become stalled or hard to heal and will need reassessment and adaptation to the initial treatment plan. Adjuvant therapies may be needed to advance the edges of the wound, such as biologic agents, negative pressure wound therapy (NPWT), skin substitutes and even closure by skin grafting or flaps. A referral to a multidisciplinary wound care team should be taken into consideration early in the management of wounds that are not progressing to healing.^17^

## Conclusion

In the ‘game of dressings’ played by many manufacturing companies, the proposed strategies can be a useful tool in guiding the primary care physician to optimise the management of all types of wounds and the indications for the adequate type of advanced dressings required.

## Recommendations for practice

- Assess the patient holistically, identify and treat the cause(s) of the wound and manage other factors that may interfere with the healing of the wound.
- Use the framework tools to prepare the wound bed.
- Choose the best available dressing taking into consideration costs, frequency of dressing changes and pain.
- Involve the patient and his or her circle of care to get adherence and compliance.
- Refer the patient to a multidisciplinary wound care team if the wound fails to heal with proper standard care.
